# Supplementary material for: Communicating Evidence about the Causes of Obesity and Support for Obesity Policies: Two Population-Based Survey Experiments
Source: Int J Environ Res Public Health. 2020 Sep 8;17(18):6539. doi: 10.3390/ijerph17186539 (PMC7559841; doi:10.3390/ijerph17186539)
Supplement: Supplementary file 1 [file ijerph-17-06539-s001.pdf]

Supplement to: **Communicating evidence about the causes of obesity and support for obesity policies in British and US populations: two population-based survey experiments**

Study 1

Messages

**Group 1.** Control	No message.
**Group 2.** Obesity (a): Availability and cost + images	Everyone makes choices about what they eat, but the food environment influences what choices are available. Currently, highly processed foods that are high in sugar and fat are easily available and much cheaper than healthier foods such as fruits and vegetables. There are also parts of Great Britain in which there is limited access to grocery stores and fresh foods, and high availability of fast food restaurants and convenience stores that sell less healthy food. Restaurant portion sizes have increased in recent years, leading people to eat more food overall, and research has indicated that food advertisements and marketing increases consumption of unhealthy foods. Therefore, aspects of the food environment play a role in causing obesity.<br>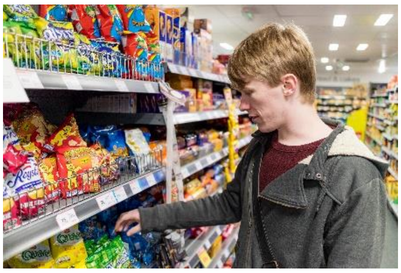 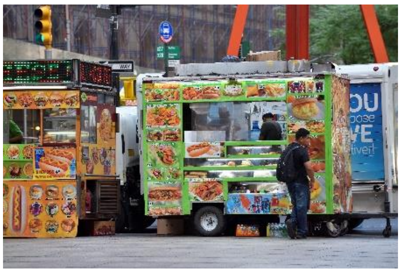
**Group 3.** Obesity (b): Cost, Availability, and marketing + images	You may think (have heard or read) that people have control over the choices that they make. For instance, you may believe that a person controls what they eat. However, the evidence suggests that this is not the complete story.<br>Everyone makes choices about what they eat but our food environments have a strong influence on this.<br>While personal choice does play a role, it only operates inside a much larger system of choices that are presented to us within our environments.<br>For example, if unhealthy food is cheaper, more widely available, and heavily marketed, research shows that people are more likely to

	choose unhealthy food.<br><br>Put simply, our food environments play a large role in causing obesity.<br><br>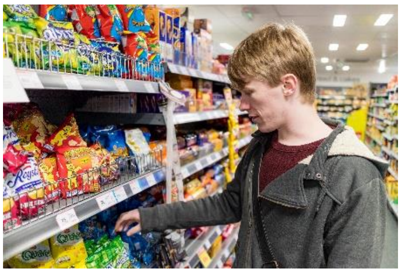 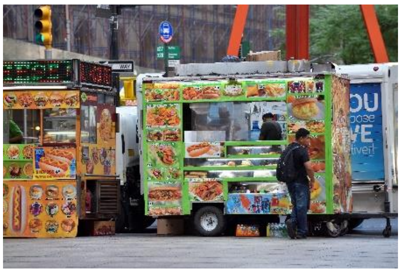
**Group 4.**<br><br>Behaviour (a):<br><br>Availability and<br><br>cost + images	Everyone makes choices about their own behaviour, but the situation and environment influences what choices are available. People only engage in the activities and behaviour that are available. In some parts of Great Britain, the options are different to those available in other parts. If people need to get to work and the only options are driving or catching a bus, then they will choose one of these two options. Consumption of single-use plastic has increased dramatically in recent years with the rise of take-away drinks and food, and pre-packaged fruit and vegetables. Therefore, aspects of the environment play a role in the general behaviour that people engage in.<br><br>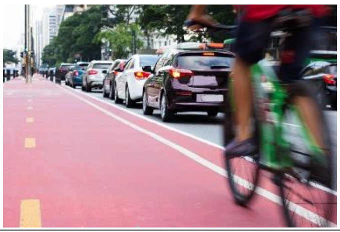 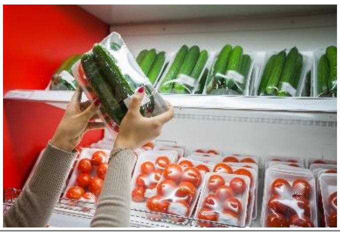
**Group 5.**<br><br>Behaviour (b):<br><br>Cost, Availability,<br><br>and marketing +<br><br>images	You may think (have heard or read) that people have control over the choices that they make. For instance, you may believe that a person is in complete control of their choice of transport or how much they recycle. However, the evidence suggests that this is not the complete story.<br><br>Everyone makes choices about what they do day-to-day but our environments have a strong influence on this.<br><br>While personal choice does play a role, it only operates inside a much larger system of choices that are presented to us within our environments.

If engaging in a given behaviour is cheaper, more widely available, and heavily marketed, research shows that people are more likely to choose this option. For example, if buying coffee in single use plastic cups is cheap and more convenient to use than multiple use plastic cups then people are more likely to use them. If there are no cycle lanes and public transport is too expensive, then people are more likely to choose driving a car despite it being less environmentally friendly.

Put simply, our environment plays a large role in our day-to-day behaviours and choices.

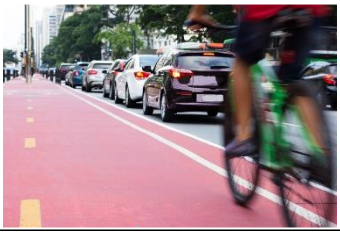

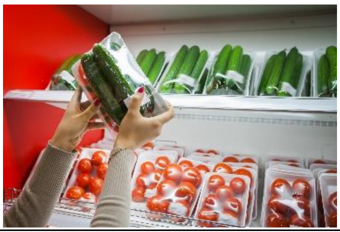

Note. The images used in Group 3 and 4 were purchased from Shutterstock and the images used in Groups 5 and 6 were purchased from IStock. All are royalty-free and we therefore have rights to use them in our research and to share in this file.

Table S1. Weighted demographic characteristics of the sample by group (%)

	Group 1: Control<br><br>(*n* = 354)	Group 2: Obesity (a)<br><br>(*n* = 312)	Group 3: Obesity (b)<br><br>(*n* = 323)	Group 4: Behaviour (a)<br><br>(*n* = 334)	Group 5: Behaviour (b)<br><br>(*n* = 358)
Gender					
Male	51	50	53	42	46
Female	49	50	47	58	54
Age					
18-24	14	11	8	15	8
25-34	13	14	12	14	10
35-44	14	16	22	18	21
45-54	17	21	21	17	21
55+	42	39	37	36	39
Socioeconomic status					
AB	28	28	27	32	25
C1C2	46	54	49	47	54
DE	25	18	24	21	21
Education					
Low	25	34	32	28	27
Moderate	44	42	42	49	39
High	31	24	26	23	34
Region					

North East	5	2	5	6	3
North West	9	10	11	11	11
Yorkshire and the Humber	8	10	10	9	11
East Midlands	7	6	6	9	8
West Midlands	10	10	10	9	8
East of England	6	10	9	7	10
London	17	11	14	13	12
South East	12	16	12	10	14
South West	11	11	11	10	13

Descriptive statistics

Table S2. Descriptive statistics (mean (SD) [*n*]) for the primary and secondary outcomes by randomised group

	Group 1: Control	Group 2: Obesity (a)	Group 3: Obesity (b)	Group 4: Behaviour (a)	Group 5: Behaviour (b)
**Support**	4.73 (1.74) [354]	4.83 (1.86) [312]	4.59 (1.88) [323]	4.72 (1.74) [334]	4.82 (1.74) [358]
**Obesity Attributions**	4.98 (1.26) [354]	4.97 (1.39) [312]	4.70 (1.36) [323]	4.89 (1.3) [334]	4.97 (1.23) [358]
**Behavioural Attributions**	5.49 (0.86) [345]	5.63 (0.91) [304]	5.44 (0.93) [315]	5.54 (0.85) [328]	5.59 (0.87) [358]
**Subjective Comprehension**	-	5.98 (0.97) [303]	5.94 (1.00) [322]	5.76 (1.04) [325]	5.98 (0.89) [347]

Sensitivity analyses (Study 1)

Covariates

The first set of sensitivity analyses were those in which there were no covariates in the models. Across most analyses, the main pattern of the results remained the same. There was no evidence that the Obesity message (a), B = -0.01, 95% CI [-0.21, 0.19], p = .891, Behaviour message (a), B = -0.10, 95% CI [-0.29, 0.10], p = .343, and Behaviour message (b), B = -0.01, 95% CI [-0.21, 0.18], p = .895, had an effect on beliefs about the environment's influence on obesity. The Obesity message (b) B = -0.28, 95% CI [-0.48, 0.09], p = .005, did significant weaken this belief, as it did in the primary analysis.

However, for one comparison there was a notable difference. In the main results, the Obesity message (a) strengthened beliefs about the environment's influence on human behaviour; however, in the model that did not include covariates, this effect was not statistically significant at the Bonferroni adjusted α = .0125 level, B = 0.15, 95% CI [0.01, 0.28], p = .037. The other three groups did not have significant effects on this belief in the primary analysis, nor in the sensitivity analyses. Obesity message (b): B = -0.05, 95% CI [-0.19, 0.08], p = .440; Behaviour message (a): B = 0.05, 95% CI [-0.08, 0.18], p = .463; Behaviour message (b): B = 0.10, 95% CI [-0.01, 0.23], p = .143.

There were no substantive differences between the primary analyses and sensitivity analyses for the policy support outcome. None of the interventions affected policy support after removing covariates from the model: Obesity message (a), B = 0.10, 95% CI [-0.18, 0.37], p = .483; Obesity message (b), B = -0.15, 95% CI [-0.42, 0.12], p = .288; Behaviour message (a), B = -0.02, 95% CI [-0.29, 0.25], p = .900; Behaviour message (b), B = 0.08, 95% CI [-0.18, 0.35], p = .542.

Outliers

The second set of sensitivity analyses were those in which outliers were not excluded. As in the primary results, there was no evidence that the Behaviour message (a), B = -0.06, 95% CI [-0.09, 0.21], p = .448, or the Behaviour message (b), B = 0.17, 95% CI [0.23, 0.31], p = .023, or the Obesity message (b), B = -0.44, 95% CI [-0.19, 0.11], p = .563, changed beliefs about environment's influence on behaviour at the α = .0125 level. The one difference was the Obesity message (a), B = 0.16, 95% CI [0.07, 0.31], p = .040, which was statistically significant at the α = .0125 level in the primary analysis but is not significant in this sensitivity analysis.

No outliers were removed from the following two variables: policy support or beliefs about environments role in obesity. Therefore, no sensitivity analyses were conducted.

Study 2

Table S3. Weighted demographic characteristics of the sample by group (%) for the USA

	Group 1: Control (*n* = 473)	Group 2: Obesity (a) (*n* = 441)	Group 3: Obesity (c) (*n* = 401)
Gender			
Male	49	45	52
Female	51	55	48
Age			
18-24	13	14	11
25-34	20	17	15
35-44	13	12	20
45-54	15	14	14
55+	38	43	39
Education			
Low	39	41	39
Medium-low	35	32	30
Medium-high	15	17	22
High	11	10	9
Region			
Northeast	18	22	19
Midwest	26	20	19
South	39	36	41
West	18	22	21

sample

Table S4. Weighted demographic characteristics of the sample by group (%) for the English sample

	Group 1: Control (*n* = 483)	Group 2: Obesity (a) (*n* = 450)	Group 3: Obesity (c) (*n* = 464)
Gender			
Male	47	47	51
Female	53	53	49
Age			
18-24	13	11	9
25-34	11	15	16
35-44	16	17	16
45-54	20	18	21
55+	40	38	37
Socioeconomic status			
AB	28	32	25
C1C2	53	47	53
DE	19	21	22
Education			
Low	29	27	34
Moderate	46	48	35
High	25	25	31
Region			
North East	5	6	6

North West	11	10	12
Yorkshire and the Humber	14	10	9
East Midlands	8	10	11
West Midlands	11	10	7
East of England	9	10	13
London	12	16	15
South East	17	16	17
South West	12	11	10

Study 2 interventions

Group 2: Obesity (a) [31]: "Everyone makes choices about what they eat, but the food environment influences what choices are available. Currently, highly processed foods that are high in sugar and fat are easily available and much cheaper than healthier foods such as fruits and vegetables. There are also parts of [America/England] in which there is limited access to grocery stores and fresh foods, and high availability of fast food restaurants and convenience stores that sell less healthy food. Restaurant portion sizes have increased in recent years, leading people to eat more food overall, and research has indicated that food advertisements and marketing increases consumption of unhealthy foods. Therefore, aspects of the food environment play a role in causing obesity."

Group 3: Obesity (c) [32]: "Lately there has been a lot of talk about the factors that influence food choices in [America/England]. For example, food advertising can lead to the selection of unhealthy food and beverages. Certain food additives, such as extra salt, sugar, and caffeine, can also increase the desire for unhealthy food. And the placement of snack food and sugary beverages at checkout counters, especially in non-grocery retail stores, can often result in unintended food purchases and overeating. Consumers should be able to make their own dietary choices. But they also need to be free from the influence of heavy advertising, exposures to habit forming food ingredients, and invasive food product placement."

Descriptive statistics

Table S5. Descriptive statistics (mean [SD]) for the primary and secondary outcomes by country and randomised group

	Group 1: Control		Group 2: Obesity (a)		Group 3: Obesity (c)	
	**England (*n* = 483)**	**USA (*n* = 473)**	**England (*n* = 450)**	**USA (*n* = 441)**	**England (*n* = 464)**	**USA (*n* = 400)**
**Support (Discouraging policies)**	0.24 (0.89)	-0.27 (0.95)	0.21 (0.95)	-0.23 (0.97)	0.20 (0.93)	-0.22 (0.99)
**Support (Encouraging policies)***	0.08 (0.84) [481]	-0.13 (0.91) [461]	0.13 (0.81) [444]	-0.05 (1.07) [430]	0.06 (0.91) [458]	0.04 (0.91) [393]
**Support for tax on confectionery**	3.38 (1.89)	2.65 (1.74)	3.52 (1.95)	2.82 (1.84)	3.34 (1.93)	2.70 (1.74)
**Support for limits on snack size**	4.18 (1.75)	3.73 (1.83)	3.97 (1.83)	3.91 (1.84)	3.98 (1.81)	3.77 (1.88)
**Support for restrictions on food ads**	5.39 (1.60)	4.25 (1.95)	5.40 (1.63)	4.23 (1.97)	5.39 (1.58)	4.60 (1.92)
**Support for more healthy food availability**	5.84 (1.19)	5.51 (1.50)	5.87 (1.25)	5.51 (1.69)	5.75 (1.34)	5.56 (1.53)
**Support for limits on SSB size**	4.61 (1.73)	3.68 (1.93)	4.47 (1.85)	3.53 (1.93)	4.56 (1.83)	3.68 (2.04)
**Support for calorie labelling on menus**	5.28 (1.50)	5.11 (1.66)	5.30 (1.46)	5.37 (1.69)	5.18 (1.57)	5.47 (1.43)

**Support for banning unhealthy snacks in schools**	4.63 (1.80)	3.85 (1.91)	4.65 (1.82)	3.96 (1.99)	4.66 (1.85)	3.99 (1.97)
**Causal belief: Food Environment**	4.79 (1.39)	4.61 (1.54)	4.67 (1.44)	4.50 (1.56)	4.59 (1.50)	4.60 (1.53)
**Causal belief: Genetics**	3.38 (1.24)	3.98 (1.34)	3.38 (1.34)	3.88 (1.35)	3.29 (1.27)	4.02 (1.31)
**Causal belief: Willpower**	4.73 (1.36)	4.51 (1.61)	4.84 (1.41)	4.57 (1.47)	4.81 (1.4)	4.60 (1.47)

Note. *Sample sizes are different for this variable and are added in [*n*]

Supplemental materials

Sensitivity analyses

Covariates

We performed sensitivity analyses to test the robustness of the main results under different analytical decisions. The first set of sensitivity analyses were those in which there were no covariates in the models. Across all analyses there were no substantive differences between the main results and these sensitivity analyses. Namely, there was no effect of the Obesity message (a), B = 0.00, 95% CI [-0.08, 0.09], p = .940, or Obesity message (c), B = 0.00, 95% CI [-0.08, 0.09], p = .955, on support for discouraging policies. There was no effect of the Obesity message (a), B = 0.07, 95% CI [-0.02, 0.15], p = .128, or Obesity message (c), B = 0.07, 95% CI [-0.02, 0.15], p = .127, on support for encouraging policies effect.

There was no effect of the Obesity message (a), B = -0.05, 95% CI [-0.17, 0.07], p = .395, or Obesity message (c), B = -0.03, 95% CI [-0.15, 0.09], p = .638, on the belief that genes cause obesity. There was also effect of the Obesity message (a), B = 0.09, 95% CI [-0.04, 0.22], p = .191, or Obesity message (c), B = 0.09, 95% CI [-0.05, 0.22], p = .199, on the belief that a lack of willpower causes obesity. And finally, there was also no effect of the Obesity message (a), B = -0.12, 95% CI [-0.25, 0.02], p = .096, or Obesity message (c), B = -0.11, 95% CI [-0.25, 0.03], p = .114, on the belief that a lack of willpower causes obesity.

Outliers

The second set of sensitivity analyses were those in which outliers were not excluded. The results of the sensitivity analyses were in accord with the primary analyses. Namely, there was no effect of Obesity message (a), B = 0.03, 95% CI [-0.06, 0.12], p = .495, or Obesity message (c), B = 0.05, 95% CI [-0.04, 0.14], p = .280, on support for Encouraging policies.

Supplemental materials

There were no outliers in the following variables: support for Discouraging policies, the belief that the environment influences obesity, the belief that genetics influence obesity, or the belief that a lack of willpower influences obesity. Sensitivity analyses were therefore not conducted for these variables.

. **Table S6.** PCA factor loadings for policy support items

	**Support for Discouraging policies (Variance explained = 52%)**	**Support for Encouraging policies (Variance explained = 14%)**
Confectionary tax	**.32**	-.14
Portion size: snacks	**.30**	-.06
Portion size: SSBs	**.29**	-.01
Advertising ban	.15	**.23**
Increase worksite healthy foods	-.02	**.50**
Menu calorie labels	-.06	**.53**
Unhealthy snack ban in schools	**.22**	.11

Note. Factor loadings over .20 are in bold.

Table S7. Interactions between group, BMI, and country on causal beliefs

	**Discouraging policies (*n* = 2586)**		**Encouraging policies (*n* = 2544)**	
	B [95% CI]	*p*	B [95% CI]	*p*
Country x Ortiz	-0.21 [-1.08, 0.67]	.645	0.19 [-0.65, 1.03]	.654
Country x P&L	-0.36 [-1.23, 0.50]	.411	-0.28 [-1.12, 0.55]	.510
BMI x country	-0.01 [-0.03, 0.02]	.555	0.02 [0.00, 0.04]	.103
BMI x Ortiz	-0.02 [-0.08, 0.03]	.396	0.00 [-0.05, 0.05]	.956
BMI x P&L	-0.04 [-0.09, 0.02]	.168	-0.01 [-0.06, 0.04]	.635
BMI x country x P&L	0.02 [-0.02, 0.05]	.331	0.01 [-0.02, 0.04]	.497
BMI x country x Ortiz	0.01 [-0.02, 0.04]	.509	0.00 [-0.03, 0.03]	.938

Note. These models control for gender, age, country, BMI, and group.

Table S8. Interactions between group, BMI, and country on causal beliefs

	**Causal beliefs: environment (*n* = 2711)**		**Causal beliefs: genetics (*n* = 2586)**		**Causal beliefs: willpower (*n* = 2711)**	
	B [95% CI]	*p*	B [95% CI]	*p*	B [95% CI]	*p*
Country x Ortiz	0.47 [-0.90, 1.83]	.504	0.29 [-0.91, 1.49]	.637	-0.78 [-2.11, 0.55]	.249
Country x P&L	0.60 [-0.75, 1.95]	.385	0.60 [-0.59, 1.78]	.325	-0.48 [-1.79, 0.83]	.471
BMI x country	0.03 [-0.01, 0.06]	.139	0.00 [-0.03, 0.03]	.909	-0.03 [-0.06, 0.01]	.112
BMI x Ortiz	0.03 [-0.05, 0.11]	.476	0.00 [-0.07, 0.07]	.982	-0.04 [-0.12, 0.04]	.293
BMI x P&L	0.03 [-0.05, 0.12]	.424	0.02 [-0.06, 0.09]	.640	-0.02 [-0.10, 0.06]	.579
BMI x country x P&L	-0.02 [-0.07, 0.03]	.382	-0.02 [-0.07, 0.02]	.307	0.01 [-0.03, 0.06]	.570
BMI x country x Ortiz	-0.01 [-0.06, 0.04]	.694	0.00 [-0.05, 0.04]	.825	0.03 [-0.02, 0.08]	.244

Note. These models control for gender, age, country, BMI, and group.

Table S9. Correlations (*Pearson's r* [95% CIs]) between policy support and causal beliefs (Study 1)

	**1.**	**2.**	**3.**
**1. Support for obesity polices**	-		
**2. Causal beliefs: Obesity**	.36 [.31, .40]	-	
**3. Causal beliefs: behaviour**	.24 [.19, .28]	.45 [.41, .50]	-

Table S10. Correlations (*Pearson's r* [95% CIs]) between policy support and causal beliefs in England and the USA (Study 2)

	**1.**	**2.**	**3.**	**4.**	**5.**
**1. Support for encouraging policies**	-	.37 [.32, .43]	.45 [.39, .50]	-.04 [-.09, .02]	.09 [.03, .14]
**2. Support for discouraging policies**	.40 [.34, .45]	-	.49 [.44, .53]	.05 [.00, .11]	-.01 [-.07, .04]
**3. Causal belief: Food environment**	.34 [.29, .39]	.49 [.45, .54]	-	.03 [-.03, .08]	.11 [.06, .16]
**4. Causal belief: Genetics**	-.07 [-.12, -.01]	.03 [-.03, .08]	.04 [-.02, .09]	-	-.08 [-.14, -.02]
**5. Causal belief: Willpower**	.14 [.09, .20]	.02 [-.03, .07]	.01 [-.04, .06]	-.16 [-.21, -.11]	-

Note. Correlations for participants residing in the USA are reported in the top right whereas correlations for participants residing in England are reported in the bottom left.
